# Supplementary material for: Synaptic plasticity and neuronal refractory time cause scaling behaviour of neuronal avalanches
Source: Sci Rep. 2016 Aug 18;6:32071. doi: 10.1038/srep32071 (PMC4989193; doi:10.1038/srep32071)
Supplement: Supplementary Information [file srep32071-s1.pdf]

Supplementary material

Synaptic plasticity and neuronal refractory time cause scaling behaviour of neuronal avalanches

L. Michiels van Kessenich[1], L. de Arcangelis[2,3], and H. J. Herrmann[1]

[1]Computational Physics for Engineering Materials, IfB, ETH Zürich
[2]Dept. of Industrial and Information Engineering, Second University of Naples, Aversa (CE), Italy
[3]INFN sez. Naples, Gr. Coll. Salerno (Italy)

Figure 4 in the paper shows that the avalanche sizes decrease when connections, and therefore loops, are added to a spanning tree. Here we discuss two effects leading to the observed reduction of avalanche sizes.

Effect 1 - The introduction of a loop reduces the size of the largest avalanche

Figure 1 shows an example configuration of a spanning tree on a 3×3 grid. For simplicity all weights are equal and the initial voltages zero, $v_i = 0$. If the stimulation occurs at the root of the tree (neuron 1), there will at some point always be an avalanche which reaches the full system size.

If a neuron has only one outgoing connection its neighbour will always fire if the parent neuron is activated. This means, neuron 7 will fire if neuron 4 fires and the chain after neuron 2 will activate in sequence if neuron 2 fires. Therefore in this configuration the neurons of interest are 1, 2 and 4. The other neurons are directly coupled to either of these three. The table below shows whether a neuron fires during different avalanches. A zero indicates inactivity and a one means that the neuron has fired during an avalanche. Neuron 1 is active in every avalanche since it is stimulated. Neuron 2 and 4 are active at every second avalanche due to the branching after neuron 1. The avalanche size is then either 1, only neuron 1 fires, or the full system, i.e. of size 9. The full system size is always reached and this is true for any system size and configuration as long as it is a spanning tree.

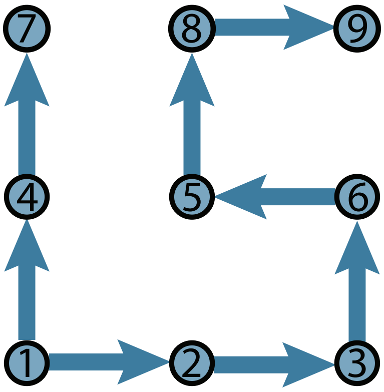

Neuron	Activity							
1	1	1	1	1	1	1	1	1
2	0	1	0	1	0	1	0	1
4	0	1	0	1	0	1	0	1
Avalanche index	1	2	3	4	5	6	7	8
**Avalanche Size**	**1**	**9**	**1**	**9**	**1**	**9**	**1**	**9**

Figure 1: Spanning tree example on a 3×3 square lattice. The table indicates whether a neuron is active during an avalanche and shows the resulting avalanche sizes. As the configuration only contains one branching the full system size is reached every second avalanche.

Now a bond is added to the configuration between neuron 5 and 2 forming a loop. The configuration in figure 2 shows the modified spanning tree. The dynamics evolving on this structure change significantly. Again stimulation occurs at the root. However, it is no longer true that all the neurons in the chain after neuron 2 fire during the same avalanches. Neurons 2,3,6,5 activate together and Neurons 8 and 9 are also coupled. Neuron 4 and 7 are still linked in the same manner as before. Therefore the neurons of interest are now 2,4 and 8. The table below shows the firing activity during different avalanches.

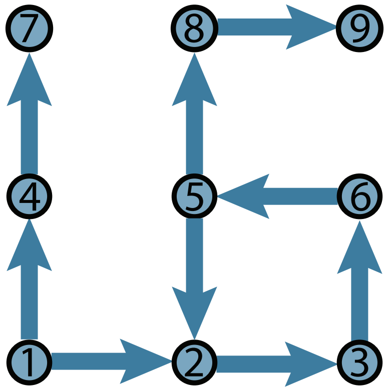

Neuron	Activity							
1	1	1	1	1	1	1	1	1
2	0	1	1	1	1	1	1	1
4	0	1	0	1	0	1	0	1
8	0	0	1	0	1	0	1	0
Avalanche index	1	2	3	4	5	6	7	8
**Avalanche Size**	**1**	**7**	**7**	**7**	**7**	**7**	**7**	**7**

Figure 2: Spanning tree with one additional bond between neuron 5 and 2 to introduce a loop. The table indicates whether a neuron is active during an avalanche and the resulting avalanche sizes.

At the first avalanche neuron 1 is triggered and gives half of the potential to 2 and 4. At the second avalanche 4 becomes active, and therefore neuron 7, as well as the chain after neuron 2 up to the branching at neuron 5. At this point neuron 2 and 8 are set to a potential of 0.5. The total avalanche size is 7 as it involves all neurons except for 8 and 9. This implies that at the next avalanche neuron 2 will be active again leading to an avalanche going through 2 all the way to 9 since neuron 8 was also at 0.5. During this avalanche neuron 4 is at zero potential and will not fire, it will go to 0.5. Because of the presence of the loop neuron 2 changes its behaviour and it will always be at a potential of 0.5 after an avalanche. It will therefore always fire since it receives 0.5 from neuron 1 at every avalanche. As a consequence, when looking at the firing pattern of neuron 8 (and 9) one can see that it will never fire during the same avalanche with 4 (and 7). It has the same firing frequency (in terms of avalanches), neuron 8 fires every second avalanche as does neuron 4, however, they are phase shifted. This means that the loop introduces a phase shift between these two branches which can never fire together. This implies that avalanches never reach the full system size. The

same effect also occurs in larger system sizes and the amount by which the largest avalanches decrease depends on the size of the branches which are phase-shifted.

Effect 2 -Where do avalanches start under random stimulation?

The effect described in the previous section clarifies why avalanches on structures with loops cannot reach the full system size. However, this argument is only valid when stimulating the root. When random stimulation is introduced one might expect to destroy the effect of phase shifting as two branches might randomly enter into phase again. Since the simulations shown in figure 4 in the paper are obtained with random stimulation we investigated this case more carefully. The network is stimulated by adding a small potential δ at random sites.

We measure where avalanches start when random stimulation is applied, i.e. which is the site that gets activated by random stimulation. This is illustrated in the figures 3 and 4. On the tree we find that most avalanches start at the root or in the upper generations of the tree. To understand this consider how a site can be discharged. It is either activated by a parent or it gets triggered by the random stimulation. Depending on the generation of a site in the tree these probabilities change. The root, for example, can only be discharged by random stimulation as it has no parents. Second generation sites have only one parent and are therefore still likely to be affected by random stimulation. Higher generation sites on the other hand have many parents and are extremely likely to be triggered by avalanches. This leads to an unequal potential distribution in the system. Low generation sites tend to have a higher average potential than high generation sites. Having a higher average potential also means a higher probability to be activated by random stimulations. Therefore randomly stimulating the network actually leads to a non-random distribution in the seed sites of avalanches. This effect occurs because of the special structure of a spanning tree. If the structure is more complex and branches meet the distribution of seed sites will again be randomly distributed. As the spanning tree is an unrealistic network for real neurons this effect is not to be expected in experimental data which indeed show a uniform distribution of initiation sites [1].

This relates to the reduction in avalanche sizes due to loops in the following way. Introducing a loop in the network will affect the site which closes the loop by increasing its average potential. The loop-closing site will never be at zero potential again as its firing will recharge it automatically (in the configuration in figure 4 neuron 2 will never be below 0.5). This means that most avalanches start now at the loop closing site as it has the highest potential. However, avalanches

starting at these sites cannot reach the largest system size since they cannot reach other branches. This implies that the probability for large avalanches decreases with each loop introduced in the spanning tree.

In conclusion, since on a spanning tree most avalanches start at the root or lower generation sites they are able to charge the branches and the system still operates much like stimulating the root. Introducing loops into the network changes this behaviour and randomly stimulating neurons will mostly trigger avalanches at loop sites which cannot lead to spanning avalanches since they are restricted to the branches to which the loop has access. Adding more loops towards the square lattice has then the effect that all sites turn into loop sites and the system becomes homogeneous. Therefore each site has equal probability to be a seed for an avalanche and furthermore each site can be reached through some path from every other site. This will then again lead to system size spanning avalanches.

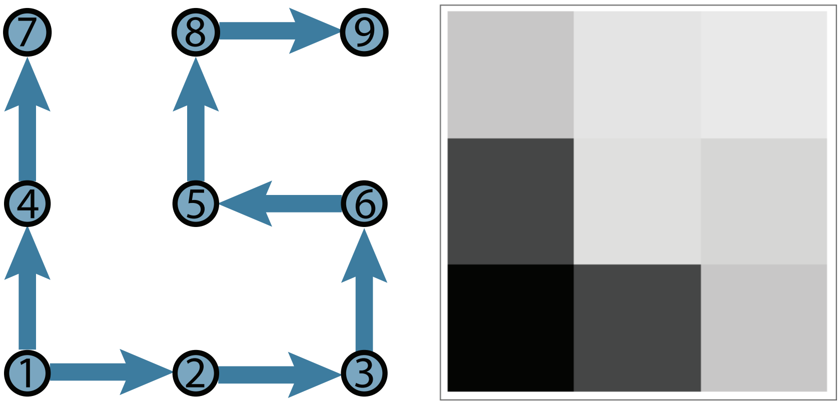

Figure 3: Where do avalanches originate? The table below shows the values plotted in the in the square on the right. A darker color means more avalanches are triggered at the corresponding site. Most avalanches start at the root. The more parents a site has, the more likely it is to be discharged by an avalanches rather than a stimulation.

0.06	0.03	0.02
0.21	0.04	0.05
0.30	0.21	0.06

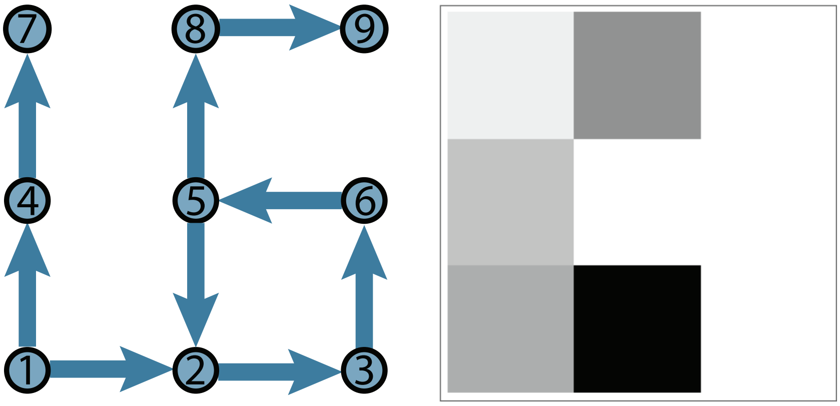

Figure 4: Introducing a loop changes the network. Most avalanches start now at the site which closes the loop as it always has a very high potential of 0.5.

0.34	0.21	0
0.11	0	0
0.16	0.49	0

References

[1] John M Beggs and Dietmar Plenz. Neuronal avalanches in neocortical circuits. *The Journal of neuroscience*, 23(35):11167–11177, 2003.
